# Interactive effects of nitrogen deposition and climate change on a globally rare forest geophyte

**DOI:** 10.1111/plb.13758

**Published:** 2024-12-25

**Authors:** B. Ohse, D. Jansen, W. Härdtle, A. Fichtner

**Affiliations:** ^1^ Ecology/Macroecology Lab, Institute of Biochemistry and Biology University of Potsdam Potsdam Germany; ^2^ Department of Vegetation Ecology and Biodiversity Conservation, Institute of Ecology Leuphana University of Lüneburg Lüneburg Germany; ^3^ Jansen & Rickert, Freelance Biologists Neumünster Germany

**Keywords:** Biodiversity, forest herb, *Gagea spathacea*, global change, interaction effects, N allocation, spring geophyte

## Abstract

- Nitrogen (N) deposition and climate change are both known to threaten global biodiversity. However, we still have a limited understanding of how interactions between these global change drivers affect individuals and populations of specialist species, such as geophytes, within their natural habitat.
- We explored possible interactive effects of N, drought, and warming on population vitality (mean leaf length, leaf density, flowering probability) and morpho‐physiological traits (e.g., leaf and bulb size, N allocation to leaves and bulbs) of the globally rare forest geophyte *Gagea spathacea* (Liliaceae) in deciduous forests of northern Germany by applying experimental N addition across a climate gradient over a 5‐year period.
- Mean leaf growth and leaf density were not affected by N addition but were enhanced by warmer and drier conditions in the months before leaf emergence. N addition increased N allocation of individual plants towards their subterranean bulbs. Importantly, effects of N addition on morpho‐physiological traits depended on warming and drought, with N‐fertilized plants showing increased leaf length and decreased specific leaf and bulb N concentration after drier autumns and warmer winters. This indicates that N deposition may partially compensate for increased N demands during warming‐induced growth, although this growth‐promoting interaction effect is not (yet) reflected in population vitality.
- Our results highlight the importance of considering multiple global environmental change drivers and a whole plant perspective (above‐ and belowground traits) to predict long‐term growth responses of (endangered) forest spring geophytes and to develop adapted long‐term protection strategies.

## Graphical abstract

Morpho‐physiological traits of leaves and subterranean bulbs of *Gagea spathacea* responded differentially to nitrogen addition and climate variation, while population vitality responded mainly to climate, thus, future population dynamics may depend on competitive interactions with more warm‐adapted and resource‐acquisitive species in the understorey.

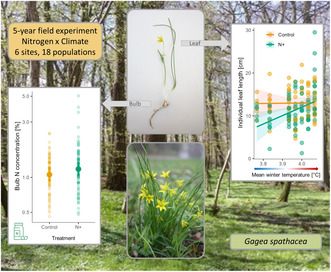

## INTRODUCTION

About 80% of the plant biodiversity in temperate forests is in the herb layer (Gilliam 2007). A characteristic feature of temperate forest herb communities are spring geophytes. These species have a distinct life history strategy with specific subterranean organs for carbohydrate and nutrient storage, such as bulbs, corms or tubers, which allow them to emerge in early spring before canopy foliation, as well as to survive unfavourable conditions during prolonged winters or summer droughts (Raunkiær 1934; Ellenberg 1988; Taylor *et al*. 2021). Geophytes are specialist species with more restricted niche breadths compared to other life forms, and with limited flexibility to respond to environmental change (Boulangeat *et al*. 2012; Lubbe *et al*. 2021). They are therefore predicted to be more threatened by rapid environmental changes and to suffer greater declines of their ranges than other life forms (Broennimann *et al*. 2006; Thuiller *et al*. 2006). Environmental variation is known to impact resource allocation to different plant parts (e.g. above‐ vs. belowground) and to different plant functions (e.g. growth, storage, or reproduction), with responses depending on the life‐history strategies of the plants (Gremer 2023). Yet, little is known so far about the interactive effects of global change drivers, such as nitrogen deposition, temperature, and drought, on resource allocation and population vitality of geophytes. To optimize conservation efforts for these specialist species, it is of vital importance to understand morphological and physiological responses of geophytes to multiple global environmental change drivers as well as associated effects on plant growth and population vitality.

Nitrogen (N) deposition is one of the most important drivers of shifts in the herb layer species composition in temperate forests (Bobbink *et al*. 2010; Bernhardt‐Römermann *et al*. 2015). N deposition can cause severe declines in the abundance of forest geophytes, such as *Anemone nemorosa* and many others (Falkengren‐Grerup 1993), and can accelerate the extinctions of small‐ranged, N‐efficient plants (Staude *et al*. 2020). Changes in N availability through increased N deposition have been linked with changes in resource allocation and, hence, plant tissue chemistry (Leith *et al*. 1999). According to the resource optimization hypothesis, additional N supply can lead to changes in N allocation patterns within plants, such that N is primarily invested into aboveground tissues (Ågren & Franklin 2003). This, in turn, may lead to increased shoot–root ratios (Friedrich *et al*. 2012; Meyer‐Grünefeldt *et al*. 2015) and allocation to flower production (Leith *et al*. 1999; Gao *et al*. 2020). However, responsiveness to N deposition differs between plant functional types (Leith *et al*. 1999), and has not yet been examined for geophytes, with their specific belowground storage organs. In order to better understand changes in plant performance and population vitality in response to global environmental changes, a detailed analysis of the effects of N addition on N allocation towards above‐ and belowground tissue, as well as towards reproductive organs of geophytes, is urgently needed.

Climate change‐related increases in temperature and drought affect forest herbs in different and partly contrasting ways. On the one hand, increasing temperatures have been shown to advance emergence time and start of flowering and have thus contributed to increased vegetative growth and reproductive success of forest geophytes (De Frenne *et al*. 2011). On the other hand, more frequent and prolonged droughts can have long‐lasting negative effects on soil moisture (Moravec *et al*. 2021), and may cause declines in the vitality of plant species adapted to moist soil conditions. How far the negative effects of water deficits on forest geophytes are compensated by positive effects of increasing temperatures remains unclear.

Interactive effects between global change drivers can severely hamper the predictive power of single‐factor studies on species responses (Zhou *et al*. 2020), and the importance of interacting global change drivers for species conservation prioritization is increasingly acknowledged (e.g. Côté *et al*. 2016). For instance, besides the observed direct effects on plant growth and reproduction, atmospheric N deposition has resulted in altered ecosystem N cycling, and has increased the amount of stored soil organic N in forests (Dörr *et al*. 2010). A larger fraction of this organic N can become available for plant growth if the predicted climate warming increases soil N mineralization rates (Rustad *et al*. 2001), such that warming might indirectly influence plant growth through its effect on N availability (De Frenne *et al*. 2011). In contrast, drought can reduce the microbial activity in temperate forests, impairing N cycling and thus inducing N shortage, but increasing N deposition could compensate for this effect under dry conditions (Dannenmann *et al*. 2016). Whether elevated plant‐available N results in altered plant growth will likely depend on how warming, drought and N deposition interactively change the resource allocation within plants. For instance, in tallgrass prairie plants, experimental warming enhanced plant N uptake and biomass production, and led to a decreased N concentration in leaf tissue (An *et al*. 2005). Increased shoot–root ratios due to N addition can also lead to higher drought sensitivity, as has been found for grasses, shrubs, and trees (Friedrich *et al*. 2012; Meyer‐Grünefeldt *et al*. 2015; Dziedek *et al*. 2016). Many forest herbs are adapted to a stable environment and thus are likely sensitive to changes in abiotic site conditions induced by N deposition and climate change. Moreover, most of them have limited dispersal ability (often <1 m yr‐1; Brunet & von Oheimb 1998) and a small range, both making their populations prone to global change and increasing the risk of extinction (Thuiller *et al*. 2005; De Frenne *et al*. 2011; Bellemare & Moeller 2014). Thus, it seems of vital importance to better understand the interactive effects of N deposition and changing weather conditions (warming, drought) on both above‐ and belowground plant traits, and therefore to improve our conservations efforts for endangered forest species in the context of global environmental change.

In this study, we used an N addition experiment across a climatic gradient to examine changes in population vitality and in morpho‐physiological traits of the spring geophyte *Gagea spathacea*. This species belongs to the Liliaceae, and is globally rare, with a distribution across northern, central, and eastern Europe, but with a large proportion of its populations being restricted to lowlands of northern Germany (Diekmann *et al*. 2014). Therefore, *G. spathacea* is one of Germany's species of special conservation responsibility and has been categorized as ‘vulnerable’ (Schnittler & Günther 1999). The bulbous geophyte emerges in early spring and senesces in late spring, with a vegetation time <10 weeks. Leaf senescence processes begin already in late March before foliation of canopy tree species. The geophyte is strongly dispersal‐limited, as it only reproduces asexually via the short‐distance dispersal of subterranean bulbils (Schnittler *et al*. 2009; Pfeiffer *et al*. 2012). *G. spathacea* thus shares key characteristics of typical forest herbs (narrow niche breadth, limited dispersal ability, small range), making the species especially prone to global environmental change. Given that *G. spathacea* has an exceptionally high N recycling rate (Fichtner *et al*. 2018) and a high N demand (Fichtner *et al*. 2020), the species might benefit from additional N input, provided that sufficient water is available for the transport of nutrients during the peak growing season in spring. Even summer droughts can have long lag effects on soil moisture in winter and spring if precipitation during autumn and winter is too low to recharge deeper soil layers, as was the case in Germany in spring 2019 after an extended summer drought in the previous year before (UFZ German Drought Monitor, based on Zink *et al*. 2016). Therefore, it seems conceivable that N deposition, warming and drought jointly affect biomass and N allocation patterns to leaves and subterranean bulbs of forest geophytes, and that such changes affect population vitality. Yet, morpho‐physiological responses of *G. spathacea* to current multiple environmental changes remain unclear, making predictions of the species' long‐term responses and adaptive management strategies difficult.

The objective of this study was to assess to what extent N addition, temperature variability and drought, as well as their potential interactions, affect *G. spathacea* at population and individual plant level. To this end, we measured population characteristics (average leaf length, leaf density, flowering probability) in N‐fertilized and unfertilized plots over a period of 5 years and related them to spatially and temporally varying regional climate data. This allowed us to assess whether *G. spathacea* becomes more sensitive to climate variability at high N deposition. Moreover, we analysed key morphological traits (leaf and bulb size and mass, bulb/leaf ratios thereof) and chemical traits (leaf and bulb N concentration and δ^13^C as indicator of drought stress) of individuals harvested after 5 years of N addition to assess the long‐term impact of N deposition and climate variability on above‐ and belowground biomass, N allocation patterns, and drought stress levels.

We hypothesized that:

1. N addition enhances population vitality and individual plant growth of *G. spathacea* and changes N allocation within plants towards the leaves.
2. Warmer winter and spring temperatures enhance population vitality and individual plant growth e.g., through earlier onset of the vegetation period, while drought stress imposed on this moist‐adapted forest herb reduces its population vitality and individual plant growth.
3. N addition is especially beneficial in combination with warmer temperatures, but is rather detrimental to population vitality and individual plant growth under drought, as N addition increases above‐ rather than belowground biomass and thus may increase plant drought sensitivity.

## METHODS

### Study area and study design

The study area is located within the core distribution area of *G. spathacea* in northern Germany (Schleswig‐Holstein). The climate of the region is sub‐oceanic, with mean annual temperatures of 8.6°C, and mean annual precipitation sums of 878 mm. In total, we investigated six populations of *G. spathacea* located in six temperate moist and nutrient‐rich deciduous forests of the alliances Alno‐Ulmion (*n* = 3), Carpinion (*n* = 2) and Fagion (*n* = 1; sensu Leuschner *et al*. 2017). Here we define populations as a collection of individuals of *G. spathacea* that occur within the same forest stand and forest community. Soil types comprised (stagnic) luvisols, stagnic gleysols and (humic) gleysols, with mull as the main humus type. The investigated forest stands have had continuous forest cover for at least 250 years, as indicated in the historical maps of Schleswig‐Holstein (Vahrendorfsche Landesaufnahme, 1789–1796). Forest stand age varied between 100 and 160 years.

**Fig. 1:**
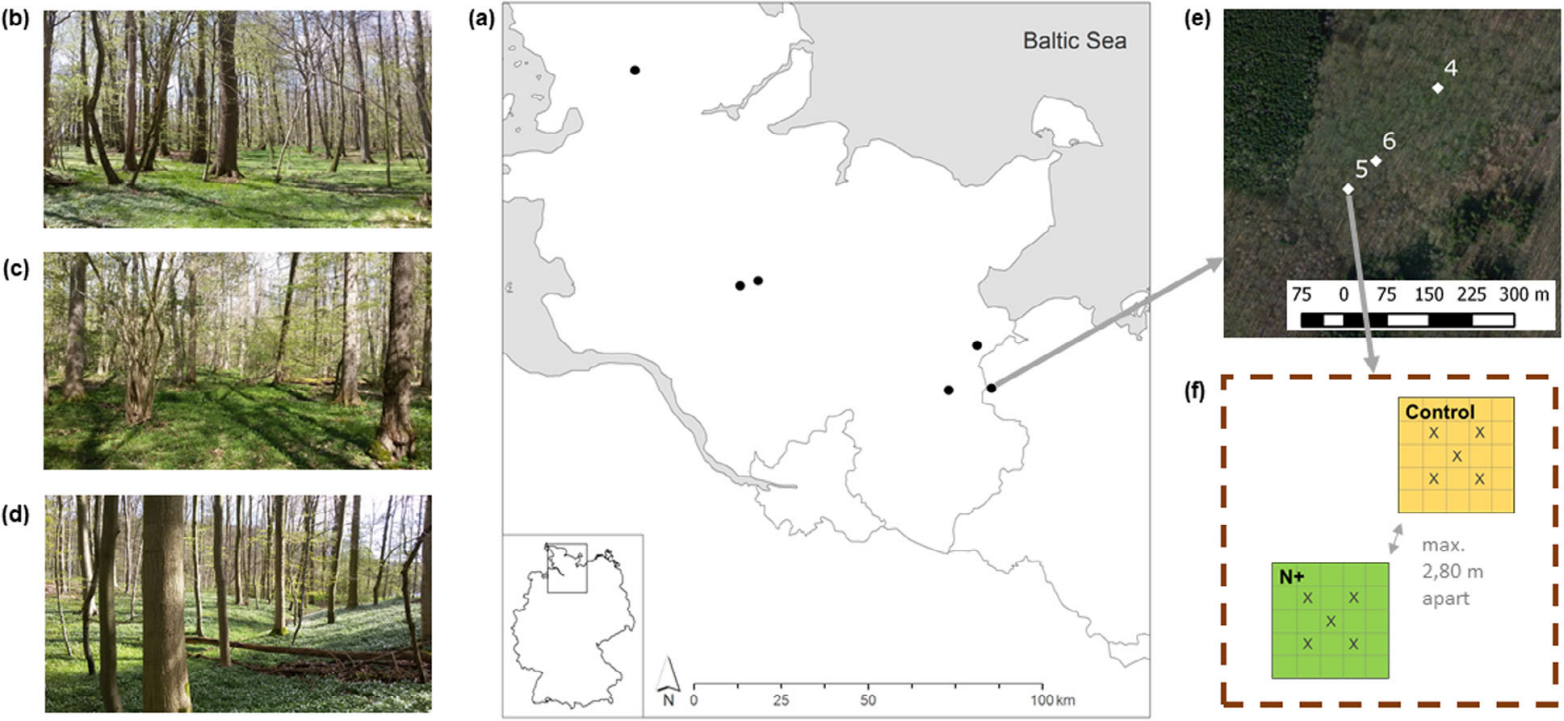
Map and study design for investigating responses of *Gagea spathacea* to climate variation and nitrogen (N) deposition. (a) Locations of the study sites across Schleswig‐Holstein, northern Germany. (b) Study forest of the alliance Carpinion. (c) Study forest of the alliance Alno‐Ulmion. (d) Study forest of the alliance Fagion (photo credits b–d: Andreas Fichtner). (e) Aerial image of a forest site with its three blocks. (f) Schematic representation of a fenced block with its two treatment plots (the 5 X in each plot mark the subplots sampled for individual traits).

In April 2014, we established a 5‐year field experiment using a randomized block design (Fig. 1). In each population, that is, at each site, we established three blocks approximately 50–150 m apart from each other. Each block was fenced to prevent disturbance and herbivory by large mammals. The blocks consisted of two 0.25 m^2^ plots (0.5 × 0.5 m) with a 1–2 m buffer to avoid edge effects and to ensure comparable site conditions. The two plots within each block were randomly assigned to an N‐treatment and a control.

The N treatment was applied each year (2015–2018), with four applications per year (early February before leaf out, early May, early July, early September). N addition was applied as ammonium nitrate, with a concentration of 40 kg ha^−1^ yr^−1^, which represents a yearly N deposition of 3.13 g per 50 cm × 50 cm plot. Due to the small amount per application date (0.78 g), ammonium nitrate was mixed with an inert carrier substrate (quartz powder, 30 g per plot) before application. A deposition rate of 20 kg ha^−1^ yr^−1^ is considered as critical load beyond which compositional changes in understorey vegetation in temperate forests can be expected (Bobbink *et al*. 2010), but this rate is exceeded in many vegetation resurvey studies across temperate forests in Europe (Bernhardt‐Römermann *et al*. 2015). Our N treatment with a deposition rate of 40 kg ha^−1^ yr^−1^ is well within the range of other N addition experiments, which have applied up to 50 kg ha^−1^ yr^−1^ (Kellner & Redbo‐Torstensson 1995; Mueller *et al*. 2013; Blondeel *et al*. 2020). However, it should be noted that only part of the experimentally added N might be available to the plants, as, for instance, the N added in autumn may not be fully available to spring geophytes in the following vegetation period due to leaching (especially during wet conditions in autumn and winter). Taken together, our treatment represents a realistic scenario for our study area, coinciding with maximum N deposition rates many understorey plants experience in forests of NW Germany.

Climate variability, especially in temperature and water availability, was captured by the spatial and temporal design of the study. More specifically, the six sites across Schleswig‐Holstein were distributed along a spatial gradient of decreasing precipitation from northwest to southeast (932–647 mm per year), while the repeated sampling across 5 years captured the temporal variation in temperature and drought (see Table S1, Fig. S1). Although an experimental manipulation of temperature and drought would have allowed us to better pinpoint mechanistic effects, experimental manipulations are much more difficult across different natural forest stands and non‐realistic if done in a greenhouse setting. Regional assessments of the soil drought status indicate a high spatial and temporal variation in soil moisture across the six sites and the five study years (see Fig. S2). We argue that our study design sufficiently captured spatiotemporal variation in climate to assess the effects of temperature and water availability on population characteristics and individual plant traits of *G. spathacea*.

### Population characteristics

To quantify changes in population vitality in response to N addition and climate variability, we recorded three main population characteristics: mean leaf length, leaf density, and flowering probability. For leaf measurements, we divided each plot (0.5 × 0.5 m) into 25 subplots (0.1 m × 0.1 m) to facilitate data collection. Leaf length, that is, the length of leaves from leaf base at soil surface to leaf tip, was measured within as many subplots (preferably within the nine central subplots) per plot as necessary to measure at least 30 leaves. From these individual measurements, mean leaf length per plot was calculated. To quantify leaf density, we counted the total number of leaves per plot. Note that some individuals can develop two leaves (in rare cases, three), which means that the number of leaves is slightly higher than the number of individuals. We also calculated the change in leaf density per plot compared to the previous year (‘Δ leaf density’) to test for lag effects in population density from one year to the next, based on the basic assumption in population ecology that the number of individuals at *t*_n_ depends on the number of individuals at *t*_n‐1_. Flowering was assessed by recording the presence or absence of flowering individuals in a plot (‘flowering probability’), as well as by counting the number of flowering individuals within plots (‘number of flowering individuals’). Population characteristics were recorded at peak vegetation period of *G. spathacea* (within less than 8 days in mid‐March) over 5 years (2015–2019). We included 2019 as the year after the last treatment to test for lag effects of N addition.

### Individual plant traits

To quantify changes in morpho‐physiological characteristics of individual plants in response to N fertilization and climate variability, individuals from each plot were harvested at the end of the experiment in March 2019, and morphological and chemical traits were analysed.

At each of the 36 plots, we collected five plant bulk samples from within the nine central subplots to avoid edge affects, ideally arranged in a quincunx (as shown in Fig. 1f). Only if there were no *G. spathacea* plants in a focal subplot or if tree roots made harvesting impossible, did we switch to the next adjacent subplot. Each plant bulk sample contained 1 to ca. 20 plants (depending on leaf density). Plant bulk samples were put into pots for transport to the lab, where individual plants were washed from the bulk samples. 22 out of 180 bulk samples contained only one individual plant. For all other bulk samples, we numbered the individuals in the bulk sample and randomly selected one number, that is, one individual plant for further analyses. Numbering and random selection of individuals was done irrespective of the individuals having one or two leaves.

Morphological traits were determined on fresh plant material. Individual leaf length was measured from the point where the leaf emerges above the bulb up to the leaf tip (for 2‐leaved plants the leaf lengths of the two single leaves were averaged to make it comparable to the leaf length of 1‐leaved plants). Bulb diameter was determined with a calliper, measured from one angle, as in *G. spathacea* bulbs are normally very round (described as ‘globose’ in Peruzzi *et al*. 2008). Leaves and bulbs were separated where the leaf emerges from the bulb, and plant parts were oven‐dried at 60°C for 48 h. Leaf and bulb dry mass were determined (for 2‐leaved plants the leaf dry mass of the two single leaves were added to make it comparable to the individual's bulb dry mass). As leaves of *G. spathacea* are small and thin and bulbs are tiny, single whole leaves and bulbs were put into tin capsules for chemical analyses. Total C and N concentrations and δ^13^C signatures of leaves and bulbs were determined using a continuous flow elemental analyser (Vario El cube; Elementar, Hanau, Germany), coupled to an isotopic ratio mass spectrometer (Isoprime IRMS; Isoprime Ltd., Cheadle Hulme, UK) (for 2‐leaved plants the values of the two single leaves were averaged). Isotope signatures were presented in the delta (δ) notation (in per mil; ‰) as a relative deviation from an international standard (Pee Dee Belemnite). The relative precision of repeated analyses of IAEA standards (IAEA‐CH‐3) was ±0.1%. Specific leaf and bulb N concentration (SLN and SBN, respectively) were calculated as N concentration [%] per unit dry mass, serving as indicators for the efficiency of N uptake to leaves and bulbs.

### Determination of soil carbon to nitrogen ratio

To account for natural differences in soil C/N ratios among forests and plots beside the experimental N addition, soil samples were taken at the end of the experiment in March 2019. Five soil samples were taken in each plot, at the exact same location as the plant samples. The soil samples represent the A_h_‐horizon, at ca. 5–10 cm depth, which is the main growing zone of bulbs and roots of *G. spathacea*. The soil samples were put into plastic bags, cooled during transport, and then oven‐dried at 105°C for 48 h. Soil samples were ground and sieved, and C and N concentration were determined using a continuous flow elemental analyser (Vario El Cube).

### Climate data

For modelling the effect of climate fluctuations on population characteristics and individual traits, we used temperature data and a 3‐month index of the Standardized Precipitation‐Evapotranspiration (SPEI). Mean monthly temperature and total monthly precipitation were extracted from the gridded climate data of the German Meteorological Service (spatial resolution 1 km × 1 km) for each of the 36 plots. Local climate variation among blocks or even plots within sites was not captured well by this medium resolution of the climate data and could thus not be explicitly included in the analyses. However, the three blocks in each site were situated close together within the same forest stand, such that local climate variation is assumed to be very low compared to the larger climate gradient across the study region and the interannual climate variation. From the extracted climate data, we calculated the 3‐month SPEI, with potential evapotranspiration being calculated according to the Thornthwaite equation (1948) (implemented in R package SPEI). The 3‐month SPEI is often used in ecological studies (e.g. Mausolf *et al*. 2018) as it has the advantage that it allows incorporation of the climate conditions of the last 3 months in the computation (e.g. important for lags in soil moisture). We used temperature (T) and SPEI of the three seasons important for the development cycle of spring geophytes (according to Lapointe 2001): spring (Mar–May), autumn (Sep–Nov), and winter (Dec–Feb). Summer was excluded as this is considered a dormant period for spring geophytes (Lapointe 2001), although it cannot be ruled out that bulbs would lose water if the soil becomes exceptionally dry. We used mean temperature and mean SPEI of the seasons preceding each measurement, as both temperature and drought potentially determine growth, resource allocation, and storage, and thereby can exert lag effects on the following year's population characteristics and individual traits. Hence, for each year of recorded population characteristics (2015–2019) we used six climate variables: T spring, T autumn, T winter, SPEI spring, SPEI autumn, SPEI winter (from each 2014–2018).

### Statistical analyses

Mixed effects models were used to analyse the effects of N fertilization and climate on population characteristics and individual traits of *G. spathacea*, while accounting for non‐independence of samples due to the spatially nested design of the study. Response variables were log‐transformed as necessary to ensure errors were normally distributed (all individual plant traits except leaf length, and leaf and bulb isotope values).

A common random effects structure was used for modelling population characteristics as well as for modelling individual traits, including nested random effects (block nested within forest site). Year was not included as a random effect, because the main differences among years consist in the climate variability among years, which is represented by the climate variables (see fixed effects below). All best‐fitting models converged with the complete random effect structure. In cases in which the model was singular with the complete random effect structure (leaf density, Δ leaf density, number of flowering individuals, bulb diameter, bulb dry mass, SLN, SBN), we excluded forest as a random effect to avoid singularity, yet, parameter estimates and test results were exactly the same as with forest included.

In most cases, linear mixed effects models were used, except for modelling leaf density, flowering probability, and number of flowering individuals, for which we used generalized linear mixed effects models with a negative binomial error distribution (leaf density and number of flowering individuals), or with binomial error distribution and logit‐link (flowering probability). Mixed effects models were set up using the R package lme4. All continuous predictor variables (temperature, SPEI, soil C/N) were centred and scaled.

As fixed effects in the full models for each of the population characteristics and individual traits, we included the N treatment in interaction with each of five climate variables (mean temperature in spring and winter, SPEI in spring, autumn, and winter) as well as the N treatment in interaction with soil C/N. We omitted mean autumn temperature, because it was highly correlated with mean spring temperature (*r* = 0.83, field data 2015–2019) and with mean winter temperature (*r* = 0.95, lab data 2019); correlations among all other climatic variables were smaller (*r* < |0.71|). For modelling individual traits, the number of leaves per individual was added as fixed factor to control for differences between plants having one or two leaves (usually emerging from daughter bulbs or mother bulbs, respectively).

To remove non‐significant terms, full models were fitted and simplified with a stepwise backward model selection. Linear mixed models were fitted with restricted maximum likelihood (REML) and model selection was based on F‐tests with Kenward‐Roger correction for degrees of freedom (lmerModlLmerTest, package lmerTest). Generalized linear mixed models were fitted with maximum likelihood (ML) and model selection was based on likelihood‐ratio tests (package lme4). Model selection results were double‐checked using parametric bootstrap for model comparison (PBmodcomp, package pbkrtest), but no differences in final variable selection were found. Thus, for each response variable a final best‐fitting model was derived containing only significant terms. Model fit was assessed by checking the normal distribution of errors using diagnostic plots. The explained variation for each response variable was assessed by calculating the marginal and conditional *R* ^2^ (rsquared, package piecewiseSEM). For all analyses, R version 4.2.3 was used.

## RESULTS

### Population characteristics

**Fig. 2:**
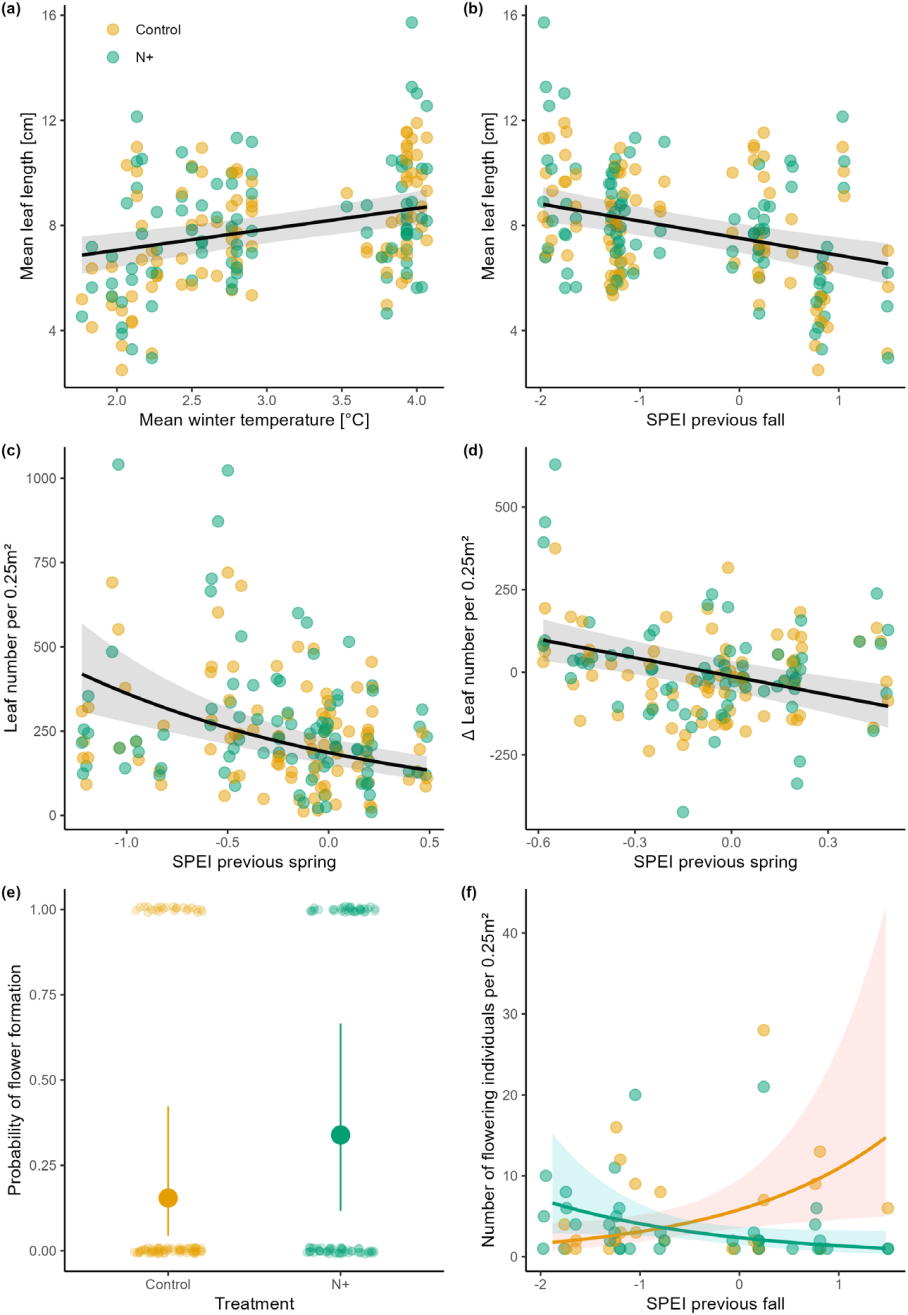
Variation in population characteristics of *Gagea spathacea* in relation to nitrogen (N) addition and climate variability (temperature and drought). (a and b) mean leaf length per plot; (c) leaf density per plot, (d) change in leaf density per plot compared to the previous year, (e) probability of flowering individuals occurring in a plot, (f) number of flowering individuals per plot. SPEI is the 3‐month mean seasonal Standardized Precipitation‐Evapotranspiration Index, with negative values indicating drier conditions than the long‐term mean of a site. Points depict the raw data values (jittered in panel e for better visibility). Large dots in (e) and lines in (a–d and f) correspond to the predicted relationships based on the best‐fitting mixed effects model (non‐target variables held at their respective mean). Bars in e and shaded areas in (a–d and f) represent the 95% confidence intervals for the fitted models. Differently coloured slopes for control and N+ indicate interaction effects between the N treatment and the respective climate variable.

Addition of N did not affect mean leaf length and leaf density (Table 1). Instead, warm winters increased mean leaf length by about 0.6 cm per °C (Fig. 2a) and increased leaf density by ca. 30 leaves per 0.25 m^2^ compared to the number of leaves in the previous year (Table 1). Furthermore, lower SPEI values (i.e., relatively dry conditions) in autumn, as well as in spring and winter, significantly enhanced mean leaf length and leaf density, respectively (Fig. 2b–d). N addition alone increased the probability of flower formation in a plot by about 10% (Fig. 2e). In contrast, the effect of N addition on the number of flowering individuals depended on climate variability. In N‐fertilized plots, the abundance of flowering individuals increased with drier autumn conditions, but the opposite pattern was evident for non‐fertilized plots (Fig. 2f).

**Table 1:** Estimates (±SE) according to best‐fitting mixed effects models of the effects of N addition and climate (temperature and drought) on population characteristics of *Gagea spathacea*.

	mean leaf length	leaf density	Δ leaf density	flowering probability	number of flowering individuals
N addition	–	–	–	1.03 ± 0.40*	−0.40 ± 0.25
Soil C/N	–	0.17 +/−0.08*	–	–	0.71 ± 0.33
T spring	–	−0.15 ± 0.05***	–	–	–
T winter	0.62 ± 0.15***	–	30.18 ± 11.21**	–	–
SPEI spring	–	−0.30 +/−0.06***	−52.19 ± 11.55***	–	–
SPEI autumn	−0.69 ± 0.15***	–	–	–	0.64 ± 0.25
SPEI winter	–	−0.20 ± 0.04***	−43.89 ± 11.75***	–	−0.43 ± 0.22
N addition × Soil C/N	–	–	–	–	−0.75 ± 0.31*
N addition × SPEI autumn	–	–	–	–	−1.20 ± 0.37***
N addition × SPEI winter	–	–	–	–	0.70 ± 0.32*
*R* ^2^_marg_	0.23	0.17	0.26	0.03	0.18
*R* ^2^_cond_	0.36	0.49	0.39	0.47	0.34
*N*	179	180	144	180	61

SPEI is the 3‐month mean seasonal Standardized Precipitation‐Evapotranspiration Index. Marginal and conditional *R* ^2^ represent model variation explained by fixed effects and the combination of fixed and random effects, respectively. Significance of test results indicated with ***P < 0.001; **P < 0.01; *P < 0.05. Main effects that were part of significant interactions were not tested separately. Variables that were not included in the final best‐fitting models of specific population characteristics are denoted by –. Δ leaf density is the change in leaf density relative to the density of the previous year.

The explained variation in most of the population characteristics ranged between 18% (number of flowering individuals), 23% (mean leaf length) and 26% (Δ leaf density) (Table 1).

### Individual plant traits

**Fig. 3:**
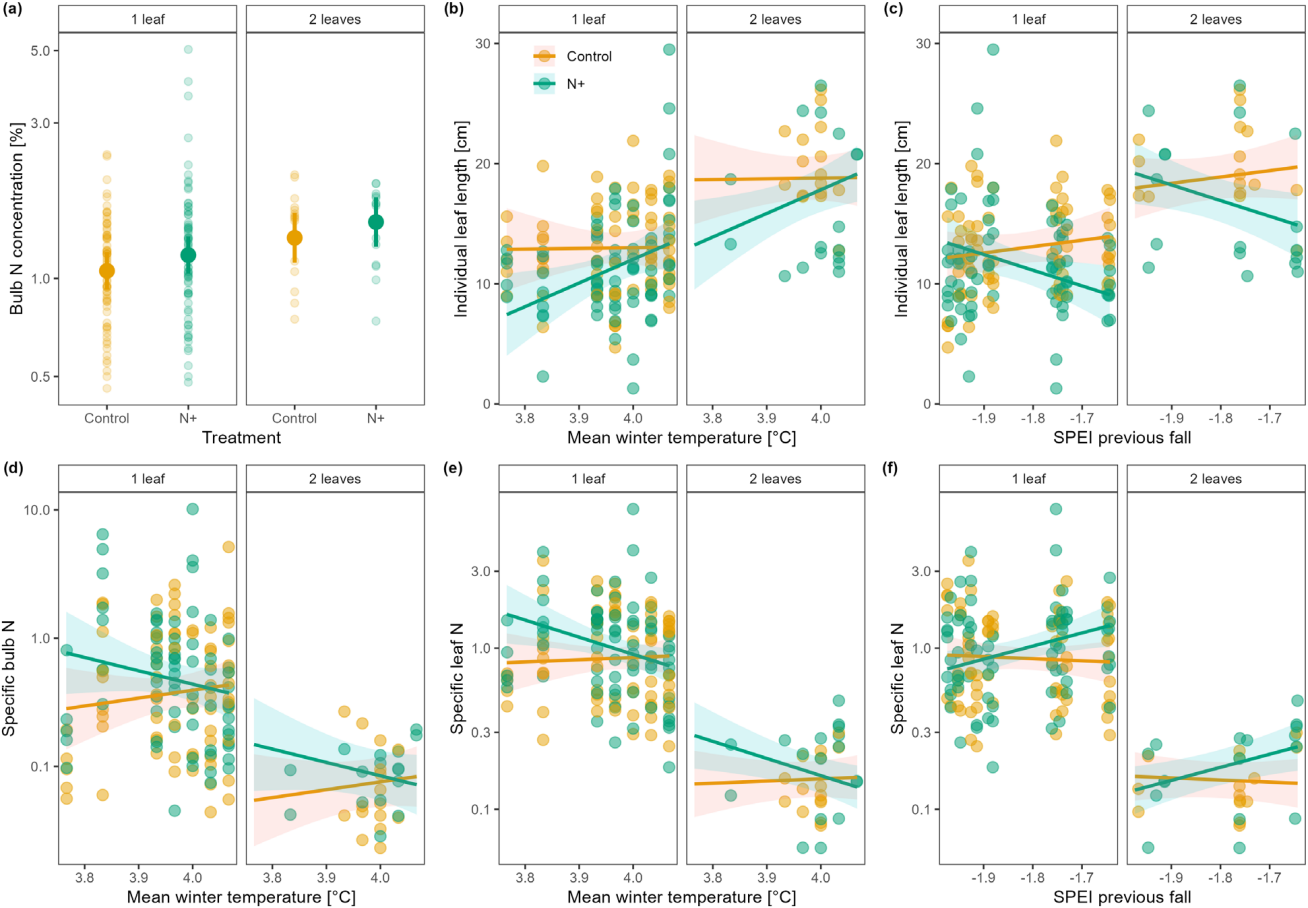
Variation in individual traits of *Gagea spathacea* with nitrogen (N) addition and climate variability (temperature and drought). (a) Bulb N concentration, (b and c) individual leaf length, (d) specific bulb N, (e and f) specific leaf N. For individuals with two leaves, the respective values of the two leaves were averaged. SPEI is the 3‐months mean seasonal Standardized Precipitation‐Evapotranspiration Index, with negative values indicating drier conditions than the long‐term mean of a site. Points depict the raw data values. Large dots in (a) and lines in (b–f) correspond to the predicted relationships based on the best‐fitting mixed effects model (non‐target variables held at their respective mean). Bars in (a) and shaded areas in (b–f) represent the 95% confidence intervals for the fitted models. Differently coloured slopes for control and N+ indicate interaction effects between the N treatment and the respective climate variable.

Overall, individual plant traits were affected by N addition, and for most of the traits (except for bulb N concentration, Fig. 3a) N effects were dependent on climate conditions (Table 2). For instance, warmer winters and drier autumns (lower SPEI values) increased individual leaf length in N‐fertilized plants by approximately 2 cm per 0.1°C and per 0.1 units of SPEI, respectively (Fig. 3b,c). Warmer winters decreased specific bulb and leaf N in fertilized plants, while drier autumns only decreased specific leaf N in the same plants (Fig. 3d–f). Interestingly, individual plant traits in control plots were largely unaffected by climate conditions (Fig. 3), indicating that N addition increases the sensitivity of plant traits to changing climate conditions.

**Table 2:** Estimates (±SE) according to best‐fitting mixed‐effects models of the effects of nitrogen (N) addition and climate (temperature and drought) on individual plant traits of *Gagea spathacea*.

	leaf length	bulb diameter	leaf dry mass	bulb dry mass	leaf N	bulb N	specific leaf N	specific bulb N	leaf δ^13^C	bulb δ^13^C	bulb/leaf size ratio	bulb/leaf N ratio
Number of leaves (2)	5.79 ± 0.81***	0.67 ± 0.05***	1.76 ± 0.12***	1.92 ± 0.17***	–	0.23 ± 0.07**	−1.73 ± 0.13***	−1.64 ± 0.21***	0.57 ± 0.14***	0.69 ± 0.16***	0.22 ± 0.06***	0.21 ± 0.06***
N addition	−1.57 ± 0.57	–	–	–	–	0.11 ± 0.05*	0.13 ± 0.09	0.23 ± 0.15	–	–	0.11 ± 0.04	0.10 ± 0.04
T winter	0.05 ± 0.68	–	–	–	–	–	0.03 ± 0.08	0.12 ± 0.14	–	–	–	0.05 ± 0.05
SPEI autumn	0.61 ± 0.68	–	–	–	–	–	−0.03 ± 0.08	–	–	–	−0.03 ± 0.06	–
N addition × SPEI autumn	−2.12 ± 0.62***	–	–	–	–	–	0.25 ± 0.10*	–	–	–	0.09 ± 0.04*	–
N addition × T winter	1.61 ± 0.62*	–	–	–	–	–	−0.23 ± 0.10*	−0.32 ± 0.15*	–	–	–	−0.08 ± 0.04*
*R* ^2^_marg_	0.31	0.48	0.55	0.43	0.00	0.07	0.54	0.29	0.07	0.08	0.13	0.10
*R* ^2^_cond_	0.39	0.51	0.57	0.48	0.27	0.35	0.58	0.40	0.41	0.38	0.30	0.33
*n*	179	179	178	178	178	176	178	176	178	172	179	175

SPEI is the 3‐month mean seasonal Standardized Precipitation‐Evapotranspiration Index. Marginal and conditional *R* ^2^ represent model variation explained by fixed effects and the combination of fixed and random effects, respectively. Significance of test results as indicated with ***P < 0.001; **P < 0.01; *P < 0.05. Main effects that were part of significant interactions were not tested separately. Soil C/N, SPEI of spring and winter, as well as spring temperature were included in the full models, but were not significant in any of the final best‐fitting mixed‐effects models, and are therefore not shown here. Variables that were not included in the final best‐fitting models of specific individual plant traits are denoted by –.

Ratios of bulb‐ and leaf‐related traits were also interactively affected by N addition and climate variability (Table 2). Specifically, the ratio of bulb to leaf size was slightly higher for N‐fertilized plants, although less dry conditions (higher SPEI values) in autumn increased the ratio of bulb to leaf size in N‐fertilized plants (Table 2, Fig. S3a). Warmer winters decreased the ratio of bulb to leaf N concentration by about 0.02 in N‐fertilized plants and increased it by about 0.02 in control plants (Fig. S3b).

Variability in most individual traits was relatively well explained by the chosen predictors (31 to 55% for size‐ and mass‐related traits, and 29%–54% for SBN and SLN). Leaf and bulb N as well as δ^13^C signatures of leaves and bulbs were poorly explained by the chosen predictors (<10%), but were largely site‐specific (indicated by very low *R* ^2^_marg_ but relatively high *R* ^2^_cond_, Table 2). Neither leaf N (Table 2), nor C:N ratios in leaves and bulbs (results not shown here) could be predicted by any of the variables measured in this study.

Most individual plant traits differed significantly between individuals with one or with two leaves (except leaf N concentration). One‐leaf plants mostly represent individuals growing from daughter bulbs, whereas two‐leaf plants often emerge from mother bulbs. However, no interaction effects between leaf number and N addition or climate variability were detected. Interestingly, N‐fertilized plots had almost double the number of two‐leaf individuals compared to control plots (42 and 22, respectively; N treatment significant with *χ* ^2^ = 6.24, df = 1, *P*‐value = 0.012, *n* = 357). A few plants with three leaves were found (4 out of 361 sampled individuals, not included in the analyses), and these only occurred in N‐fertilized plots.

## DISCUSSION

Contrary to our expectations, N addition did not enhance population vitality, that is, mean leaf growth and leaf density, but increased flower formation and N allocation of individual plants towards their subterranean bulbs. As hypothesized, warmer winter temperatures enhanced population vitality. Surprisingly, dry conditions in the previous spring and winter did not increase drought stress in plants, but rather contributed to enhanced population vitality. Most importantly, N addition interacted significantly with warming and drought to increase individual leaf growth and to change N allocation from leaves towards bulbs.

### Nitrogen addition does not affect population vitality but promotes surplus flowering

Responses of leaf growth and leaf density to N addition seem to be very species‐specific (Kellner & Redbo‐Torstensson 1995; Rainey *et al*. 1999; Siefert & Ritchie 2016; Blondeel *et al*. 2020) and data for geophytes are rare. We found no response of mean leaf length and leaf density to N addition in our study, although *G. spathacea* has a high N demand and high N recycling rates (Fichtner *et al*. 2018, 2020) and was thus expected to be rather responsive to N addition. Instead of enhancing plant growth, supplied nutrients could have been lost to microbial immobilization (Zak *et al*. 1990; Aerts & Chapin 1999), as soil microorganisms can take up eight times more N than spring ephemerals (Rothstein 2000) and can do so faster than plants (Andresen *et al*. 2008), thus obscuring plant responses to N addition. Variation in leaf density of *G. spathacea* can be high, ranging in our study from 10 to 1041 leaves per 0.25m^2^ (see summary statistics in Table S2), although it is unrelated to the other population characteristics measured in this study (Fig. S4). Leaf density was not driven by N addition, but was largely explained by the specific forest stand (indicated by a high conditional *R* ^2^ relative to the marginal *R* ^2^). Leaf density was partly (but not significantly) explained by the soil C/N ratio, which varied mostly among forest sites, but not between treatments (results not shown). This is in line with findings from a previous observational study with 40 populations of *G. spathacea* across different forest communities that showed slightly lower soil C/N ratios in Alno‐Ulmion forests (Fichtner *et al*. 2020). As dispersal processes of this species are mainly stochastic and short‐distance via translocation of bulbils through uprooting or animals (Schnittler *et al*. 2009; Pfeiffer *et al*. 2012), it can be assumed, that leaf density of *G. spathacea* populations strongly depends on the time period for dispersal, that is the long‐term presence of a population (Fichtner *et al*. 2018). Stand‐specific population densities thus highlight the importance of forest habitat continuity for ensuring population vitality of this geophyte (Fichtner *et al*. 2020) as well as of many other geophytes that face similarly limited dispersal capabilities.

Flowering can be highly sensitive to N deposition, with both increases and declines seen in dominant shrub and forb species, respectively (Phoenix *et al*. 2012). Flowering of *G. spathacea* was enhanced in our study, corroborating findings from observations across different forest communities showing increased probability of flowering of *G. spathacea* at sites with higher soil N concentration (Fichtner *et al*. 2020). It is also in line with our observation that flowering only occurs in *G. spathacea* individuals with two or three leaves, which we found to be significantly more abundant in N‐fertilized than in unfertilized plots. Interestingly, N addition promoted flowering in *G. spathacea*, even though flowering does not contribute to population dynamics, as the species only reproduces asexually through translocation of its subterranean bulbils (Pfeiffer *et al*. 2011). On the contrary, the energetic cost of increased flowering due to increased N deposition might have negative consequences for growth and future survival. However, in *G. spathacea*, N allocation to flowers is generally low (6%) compared to the closely related *Gagea lutea* (15%), while N allocation to daughter bulbs is three times higher than to flower formation in *G. spathacea* (Schnittler *et al*. 2009). Increased probability of flowering due to ample resource availability is also known from other (clonal) geophytes (Verboom *et al*. 2002). Hence, when analysing trade‐offs in resource allocation of geophytes, for example, to growth vs. reproduction, the availability of N as well as the main mode of reproduction (sexually vs. asexually) needs to be accounted for. The relatively high conditional *R* ^2^ (reflecting the specific forest stand) could indicate an influence of other environmental factors, such as soil moisture, which can vary significantly between forest stands (Fichtner *et al*. 2020).

### Warmer conditions might enhance population vitality, depending on soil moisture

Warmer winter and spring temperatures can benefit early spring geophytes by advancing their phenology (Petrauski *et al*. 2019). We indeed found significant positive effects of warmer winters on mean leaf growth of *G. spathacea*, with enhanced leaf growth indicating improved vitality of the species (Schnittler *et al*. 2009). This finding corroborates results from recent studies on three other geophytes in temperate forests, that also found increased plant height with warmer winters (Vangansbeke *et al*. 2022). But if trees leaf out earlier, which can be the case with warmer spring temperatures (Montgomery *et al*. 2020), the growing season of spring geophytes may be terminated earlier due to light limitation, such that spring ephemerals might be forced to complete their epigeous growth period more quickly in a warmer climate (Lapointe 2001). This is already the case in some North American temperate forests, where overstorey tree leaf out is more responsive to warmer spring temperatures than is the phenology of understorey wildflowers (Heberling *et al*. 2019). So far, *G. spathacea* ends its aboveground developmental cycle before foliation of the canopy tree species, that is, when the effect of the canopy cover on understorey temperature and light conditions is still minimal (Sanczuk *et al*. 2023). However, whether *G. spathacea* and other forest spring geophytes will benefit from warmer winter and spring conditions in the long term will likely depend on the geophytes' growing season length relative to the leaf‐out dates of the overstorey tree species (Lapointe 2001; Montgomery *et al*. 2020; Lee *et al*. 2024). Some geophytes only profit from winter warming at the start of their growing season, but are negatively impacted in their height growth later on (Vangansbeke *et al*. 2022). Hence, long‐term population trends of geophytes might also depend on changes in the competitive strength of geophytes compared to other, more warm‐adapted life forms that emerge later in spring (Lubbe *et al*. 2021).

Climate warming can increase drought stress in many forest herbs (Svenning & Skov 2006). In contrast, *G. spathacea* individuals did not show any signs of drought stress, as indicated by δ^13^C signatures (see Table 2). Instead, drier conditions (i.e., more negative SPEI values) enhanced mean leaf growth and leaf density. On the one hand, the adaptation of geophytes to frost is often accompanied by adaptation to drought (Fang *et al*. 2023), which would explain missing signals in δ^13^C signatures. On the other hand, prolonged and severe droughts can force buds into long‐term dormancy that exceed their natural longevity (Ott *et al*. 2019). In natural ash and alder‐ash forests – the main habitat of *G. spathacea* (Fichtner *et al*. 2020) – soil water deficits are less conceivable. In exceptionally wet winters, these forests can be partly flooded. A drier autumn/winter could thus lead to reduced flooding or an earlier point of drying out, and could thus result in an earlier onset or a prolongation of the growing season for *G. spathacea* and other forest spring geophytes. This may explain the positive response of the geophyte's population vitality to dry conditions (low SPEI values) in our study. On the other hand, *G. spathacea* has been observed to grow more vigorously in the moist Alno‐Ulmion forests than in the significantly drier Fagion or Carpinion forests (Fichtner *et al*. 2020). However, management‐related drainage of moist forests, combined with prolonged summer drought events, could further decrease long‐term soil water supply in the future and could potentially subject *G. spathacea* and other forest geophytes to drought stress. Unfortunately, experimental drought on small spatial scales does not necessarily result in a concurrent soil water decrease in the forest understorey (Herberich *et al*. 2023). Hence, to better understand the interplay of increasing temperature, N deposition and changes in soil moisture and their combined effects on the long‐term population dynamics of spring geophytes, we advocate an approach similar to that presented in this study, but with plot‐specific documentation of temporal variations in soil moisture.

### Nitrogen addition and climate variability interactively affect individual plant growth and N allocation towards bulbs

Interaction effects of temperature and the global change driver CO_2_ on plant biomass are relatively consistent (Dieleman *et al*. 2012), yet, evidence for interaction effects of temperature and N addition on individual plant traits is limited (Blondeel *et al*. 2019, 2020). We expected N addition to be beneficial, especially in combination with warmer temperatures, as N addition can increase foliar N in forest herbs (Gilliam 2006), while warming can enhance plant N uptake and biomass production (An *et al*. 2005). Interestingly, we found that individual leaf length increased in warmer winters especially in N‐fertilized plants, while leaf N concentration was unchanged. Our results thus indicate that N addition might ‘compensate’ for the increased N uptake during warmer conditions, such that leaf length of N‐fertilized plants could increase, while leaf N concentration stays the same. This seems to be in contrast to other recent global change interaction experiments that found no interaction effects of N addition and warming on plant growth or leaf N content (Blondeel *et al*. 2019, 2020). However, the same experiment found that warming decreased the leaf N content, especially in slow‐colonizing, N‐conserving forest herbs, implying a tissue dilution of N with increasing growth (Blondeel *et al*. 2019). Our results suggest that this dilution effect might be compensated by further N addition in some species. Whether such an N compensation mechanism through N addition is a more general mechanism in N conservative species, or whether it is specific to geophytes warrants further investigation.

Another effect of increased N supply (beyond N saturation) can be enhanced NO_3_^−^ production and mobility and high lateral transport of unbound NO_3_^−^, especially in water‐saturated soils, leading to lateral losses and thus decreased availability of essential soil cations, such as Ca^2+^ and Mg^2+^ to herb layer species (Gilliam 2006; Calvo‐Fernández *et al*. 2015). N deposition in the study region Schleswig‐Holstein is comparably high (>20 kg N ha^−1^ year^−1^, Schaap *et al*. 2018) and soils at the study sites can occasionally be waterlogged during winter. Together, this might explain why we found unexpected negative effects of N addition on individual leaf length under wet conditions. These negative effects of N addition were only detected for total individual leaf length, that is, comprising both the above‐ and below‐ground parts of the leaves as measured in the lab. The field measurements, comprising only the aboveground part of the leaves seem to not capture this effect of N addition (see also Fig. S5), either because of undetected effects of N on the belowground part or because of a larger measurement error in the field.

We consider that the net effect of increased N availability and decreased base cation availability on leaf growth will strongly depend on the future climate water balance in these forest ecosystems. It should be noted though, that for N and base supply, the litter quality of the overstorey trees can be a stronger driver of topsoil conditions compared to atmospheric deposition, with high litter quality providing soils with a higher proportion of exchangeable base cations (Maes *et al*. 2019). This might also be the case for *G. spathacea* and co‐occurring forest geophytes (e.g. *A. nemorosa* and *Ficaria verna*), which benefit from the presence of *Fraxinus excelsior* (European ash) trees in the overstorey due to high‐quality litter (Fichtner *et al*. 2020). However, recent ash dieback disease (Coker *et al*. 2019) may result in profound changes in forest structure and overstorey tree species composition in formerly ash‐dominated forests (Needham *et al*. 2016). Associated changes in understorey light availability and decreased litter quality would likely lead to competitive exclusion by more resource acquisitive generalist species, such as *Rubus fruticosus* agg. Thus, forest conservation and management that help preserve ash‐dominated forests could contribute significantly to the long‐term vitality of *G. spathacea* populations and its typical co‐occurring herb species.

Allocation of N towards belowground organs of forest geophytes in response to global change has, to our knowledge, not yet been studied in depth. Filling this gap, an important finding of our study was that bulb size was not affected by N addition but that bulb N concentration significantly increased, indicating that surplus N is not used for bulb growth but is accumulated in the bulbs. Accordingly, ratios of bulb to leaf N concentration were higher in N‐fertilized plants. Interestingly, this ratio of bulb to leaf N decreased in mild winters, pointing to a relocation of bulb N to be invested into extended leaf growth under warmer conditions. Spring ephemerals are known to have a higher nutrient resorption efficiency than other forest herbs (Lapointe 2001). However, significant decreases in *G. spathacea* specific bulb and leaf N (i.e., N concentration relative to overall bulb or leaf biomass) and a decrease in the ratio of bulb to leaf N concentration in fertilized individuals during warmer winters suggest complex dependencies of N allocation between above‐ and belowground organs of geophytes on the overall N cycle. Eventually, continued warming and N deposition could increase the competition from non‐geophytes, as for example, graminoids seem to respond more plastically to N and temperature increases than forbs (De Frenne *et al*. 2011), making future changes in the forest herb (functional) community composition more likely. Our results strongly indicate that global change drivers need to be considered jointly and that considering N allocation to belowground storage organs is essential for assessing the overall vitality of geophytes under global change.

## CONCLUSIONS

By examining the combined effects of N deposition and climate conditions on population vitality and above‐ and belowground traits of *G. spathacea*, this study enhances our understanding of how global change drivers interactively affect resource allocation and population dynamics of spring geophytes. According to our results, plant individuals seem to benefit from a combined effect of warming and N addition. Surprisingly, effects of N addition were not reflected in changes in population vitality. Thus, more long‐term field experiments involving interaction effects of warming, soil moisture availability, and N deposition will be needed to better understand how effects on N allocation and biomass of above‐ and belowground organs translate into future population dynamics of geophytes. A more systematic investigation of resource allocation to belowground storage organs of forest geophytes would also help reveal differences in the resilience of geophytes versus non‐geophytes to global change drivers. Lastly, our study underlines that population dynamics of forest geophytes with low dispersal power might strongly depend on site history and habitat continuity, hence, preserving extant populations and their habitat is paramount.

## AUTHOR CONTRIBUTIONS

WH and AF conceived and designed the experiment; AF, DJ and BO conducted fieldwork; BO performed the laboratory work and analysed the data. BO wrote the first draft of the manuscript; all authors reviewed and edited the manuscript.

## CONFLICT OF INTEREST STATEMENT

The authors declare that they have no conflict of interest.

## Supporting information


Data S1.
