# Supplementary material for: Interactive effects of nitrogen deposition and climate change on a globally rare forest geophyte
Source: Plant Biol (Stuttg). 2024 Dec 25;27(2):297–309. doi: 10.1111/plb.13758 (PMC11846631; doi:10.1111/plb.13758)
Supplement: Supplementary file 1 — Data S1. [file PLB-27-297-s001.docx]

# Supporting Information

Table S1 Spatial variation in climate across the study region. Given is the mean annual precipitation sum and the mean annual temperature across the study period 2015-2019. Values were calculated from the gridded monthly data of the German Meteorological Service. Sites are ordered following a spatial gradient of decreasing precipitation from northwest to southeast.

| **Forest site** | **Precipitation sum [mm]** | **Temperature [°C]** | **Latitude** | **Longitude** |
| --- | --- | --- | --- | --- |
| *Pobuell* | 932 | 9,5 | 54,6168 | 9,2449 |
| *Meezen* | 863 | 9,5 | 54,0593 | 9,7025 |
| *Hasselbusch* | 847 | 9,6 | 54,0712 | 9,7811 |
| *Schellbruch* | 670 | 9,8 | 53,8946 | 10,7364 |
| *Kannenbruch* | 651 | 9,7 | 53,7810 | 10,6094 |
| *Schattin* | 647 | 9,5 | 53,7836 | 10,7948 |


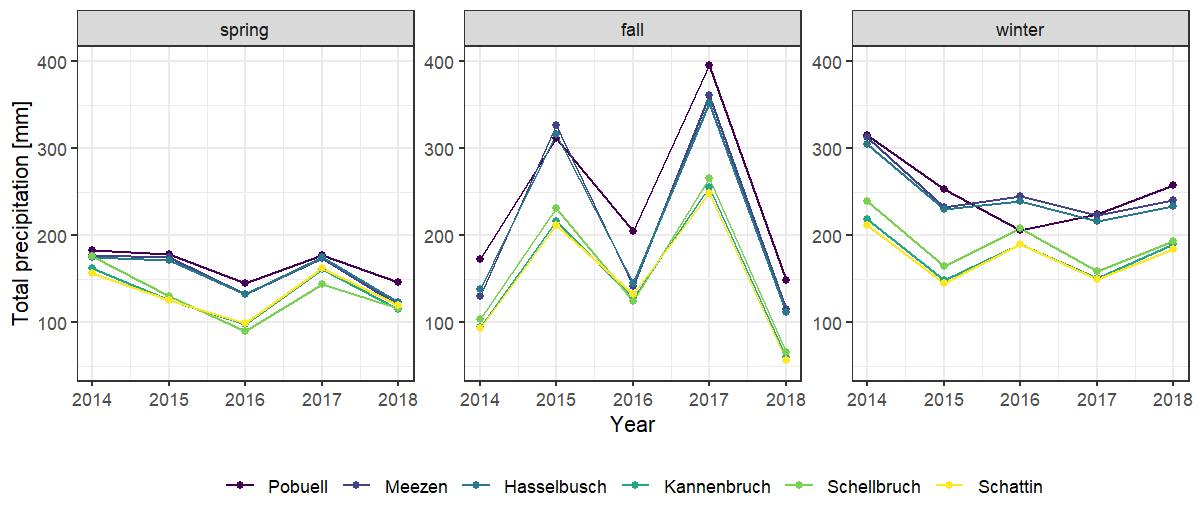

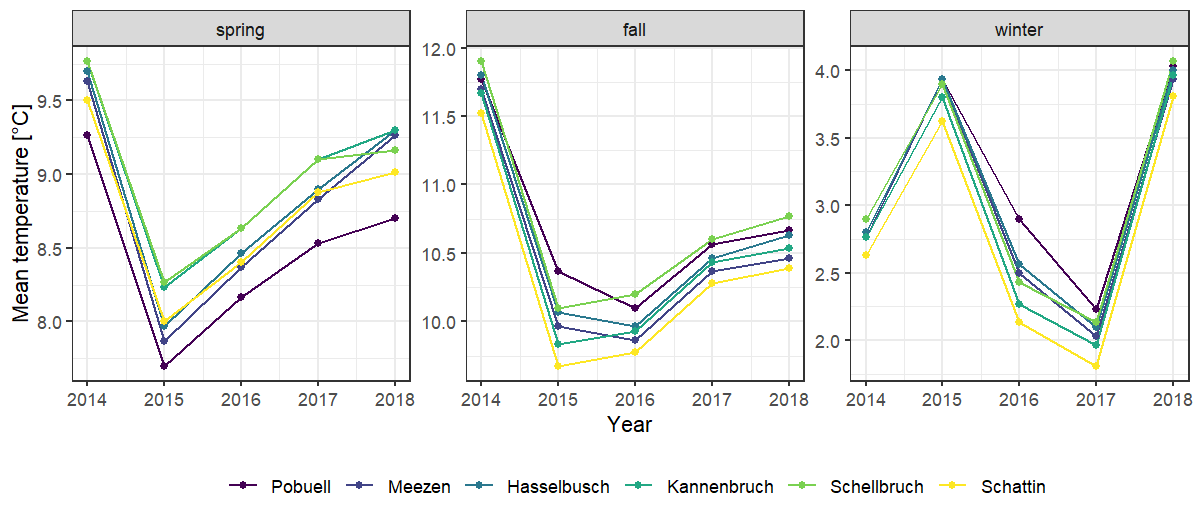

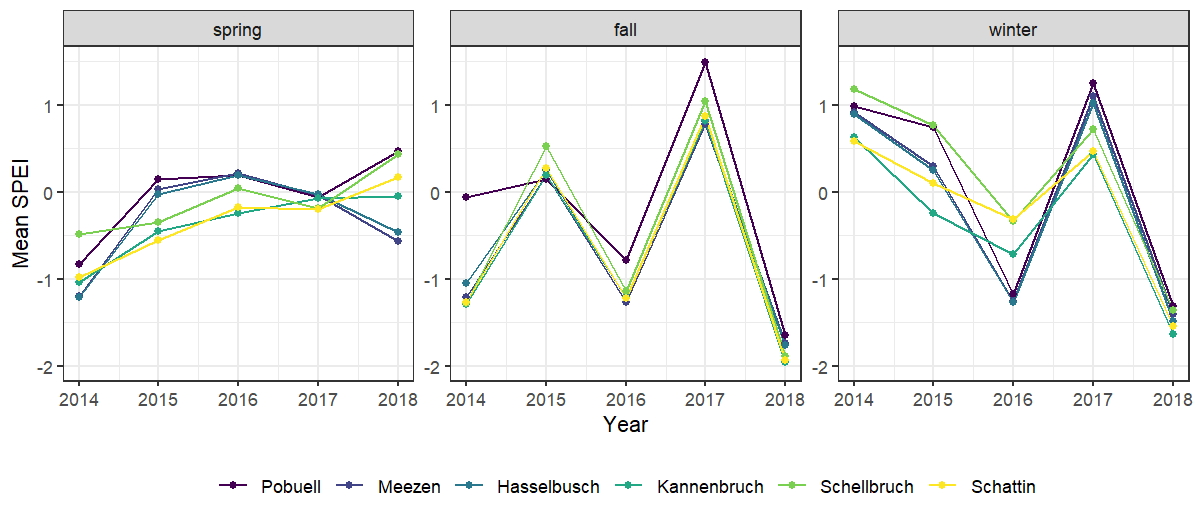


Figure S1 Temporal variation in climatic conditions in each of the six sites across the study period. Colors indicate the sites’ position along the spatial gradient of decreasing precipitation from northwest (site Pobuell) to southeast (site Schattin). Values are given per season (spring – Mar-May, fall – Sep-Nov, winter – Dec-Feb), each in the year prior to growth of *Gagea spathacea*. Note different y-axis limits for mean temperature in the different seasons.


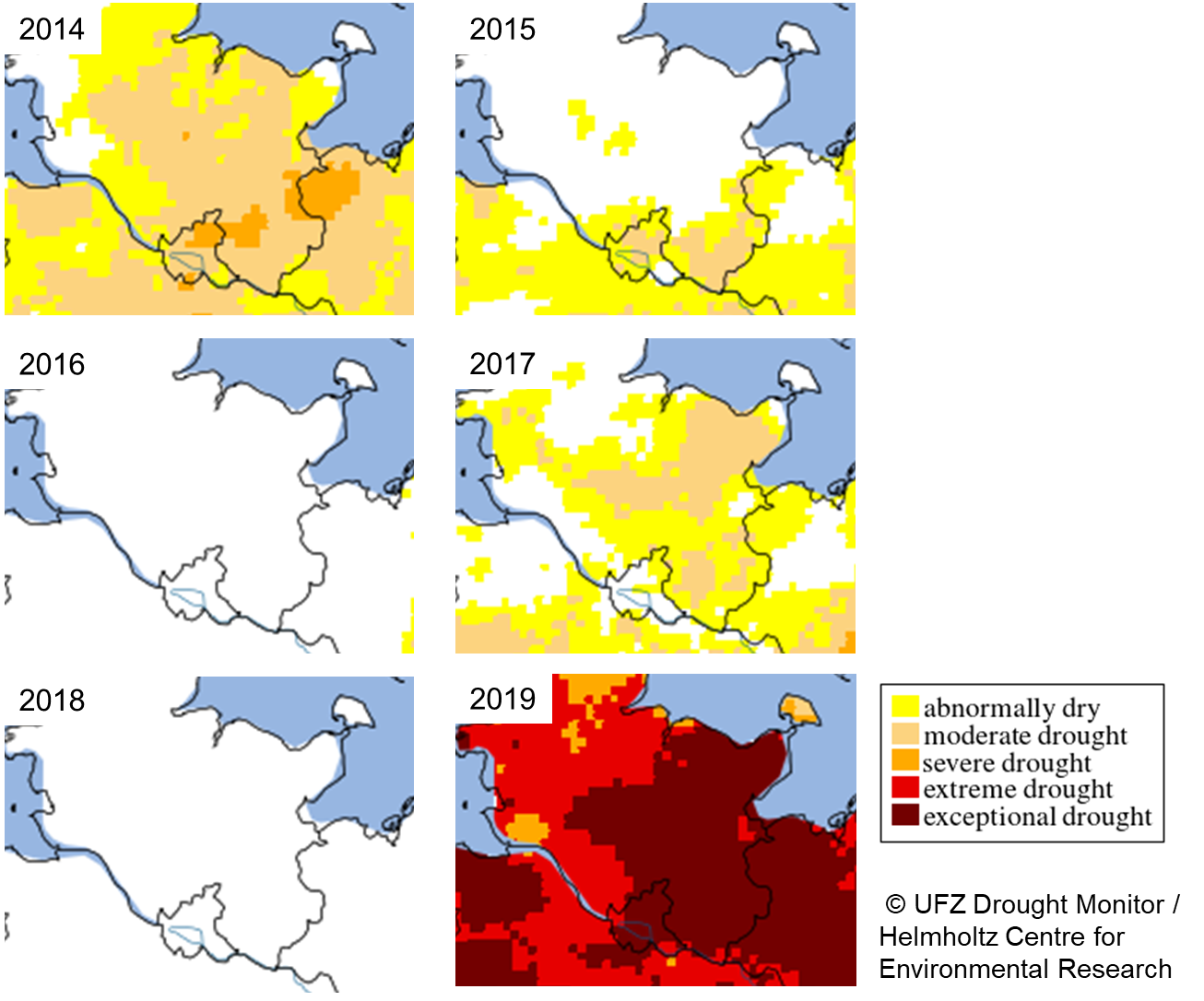


Figure S2 Spatial and temporal variation in climatic conditions across the study region in Schleswig-Holstein, Northern Germany. Shown is the drought status in the total soil column (down to 1.80 m) in March (main growing season of *Gagea spathacea*) of each year. The drought status generally increases from the north-western to the south-eastern part of the study region and shows a large variation across years. Figures are taken from the Drought Monitor of the UFZ-Helmholtz Centre of Environmental Research.

Drought classes are based on the soil moisture index (SMI, Samaniego et al., 2013), which is calculated on the basis of the mesoscale Hydrological Model (mHM, [www.ufz.de/mhm](http://www.ufz.de/mhm)) and shows the soil moisture distribution over the period 1951-2015.

SMI 0,20 - 0,30 = abnormally dry

SMI 0,10 - 0,20 = moderate drought

SMI 0,05 - 0,10 = severe drought

SMI 0,02 - 0,05 = extreme drought

SMI 0,00 - 0,02 = exceptional drought

A value of 0.3 (abnormally dry) means that the current soil moisture is as low as in 30% of the cases from 1951–2015. A description of the model can be found in Zink et al. (2016). The calculations of the SMI are performed on the basis of the operational hydrological modelling using the mHM.

Samaniego, L., Kumar, R., & Zink, M. (2013). Implications of Parameter Uncertainty on Soil Moisture Drought Analysis in Germany. *Journal of Hydrometeorology*, *14*(1), 47–68.

Zink, M., Samaniego, L., Kumar, R., Thober, S., Mai, J., Schäfer, D., & Marx, A. (2016). The German drought monitor. *Environmental Research Letters*, *11*(7), 074002.


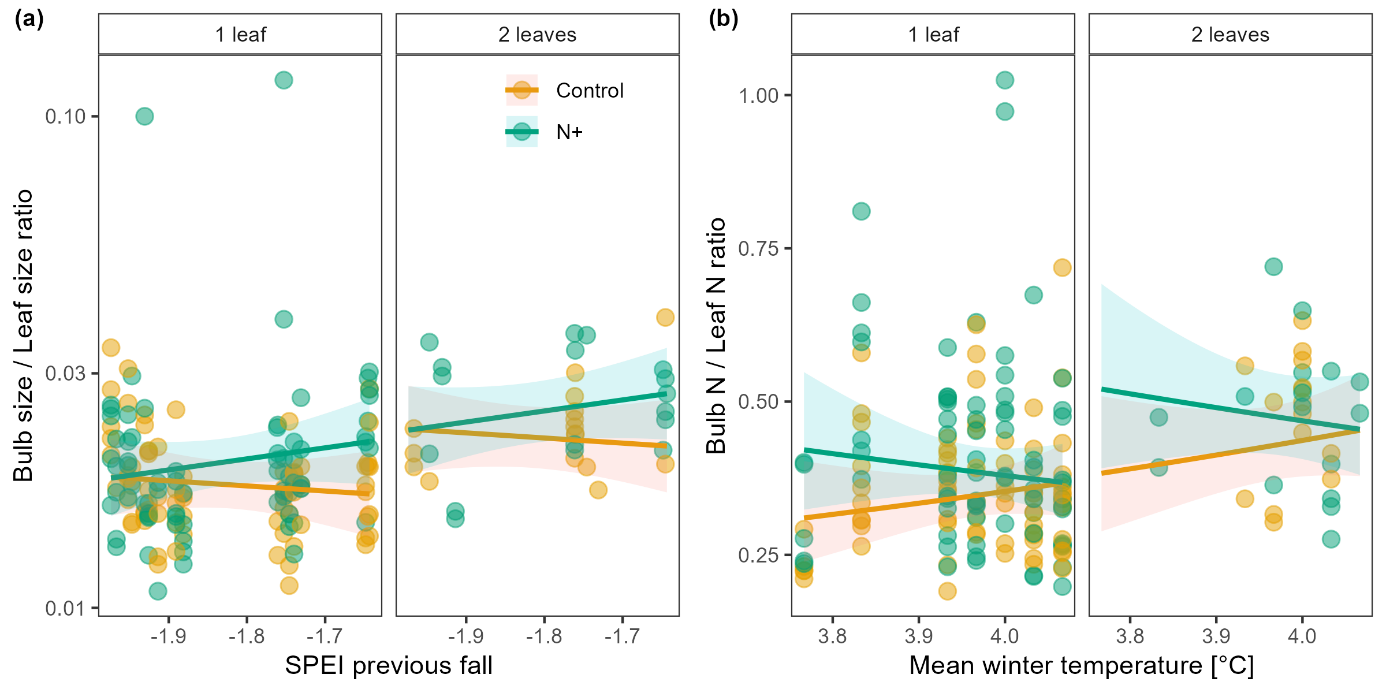


Figure S3 Variation in individual traits of *Gagea spathacea* with nitrogen (N) addition and climate variability (temperature and drought). **a** ratio of bulb diameter to leaf length, **b** ratio of bulb to leaf N concentration. For individuals with two leaves, the respective values of the two leaves were averaged. SPEI is the 3-months mean seasonal Standardized Precipitation-Evapotranspiration Index, with negative values indicating drier conditions than the long-term mean of a site. Points depict the raw data values. Lines correspond to the predicted relationships based on the best-fitting mixed effects model (non-target variables held at their respective mean).


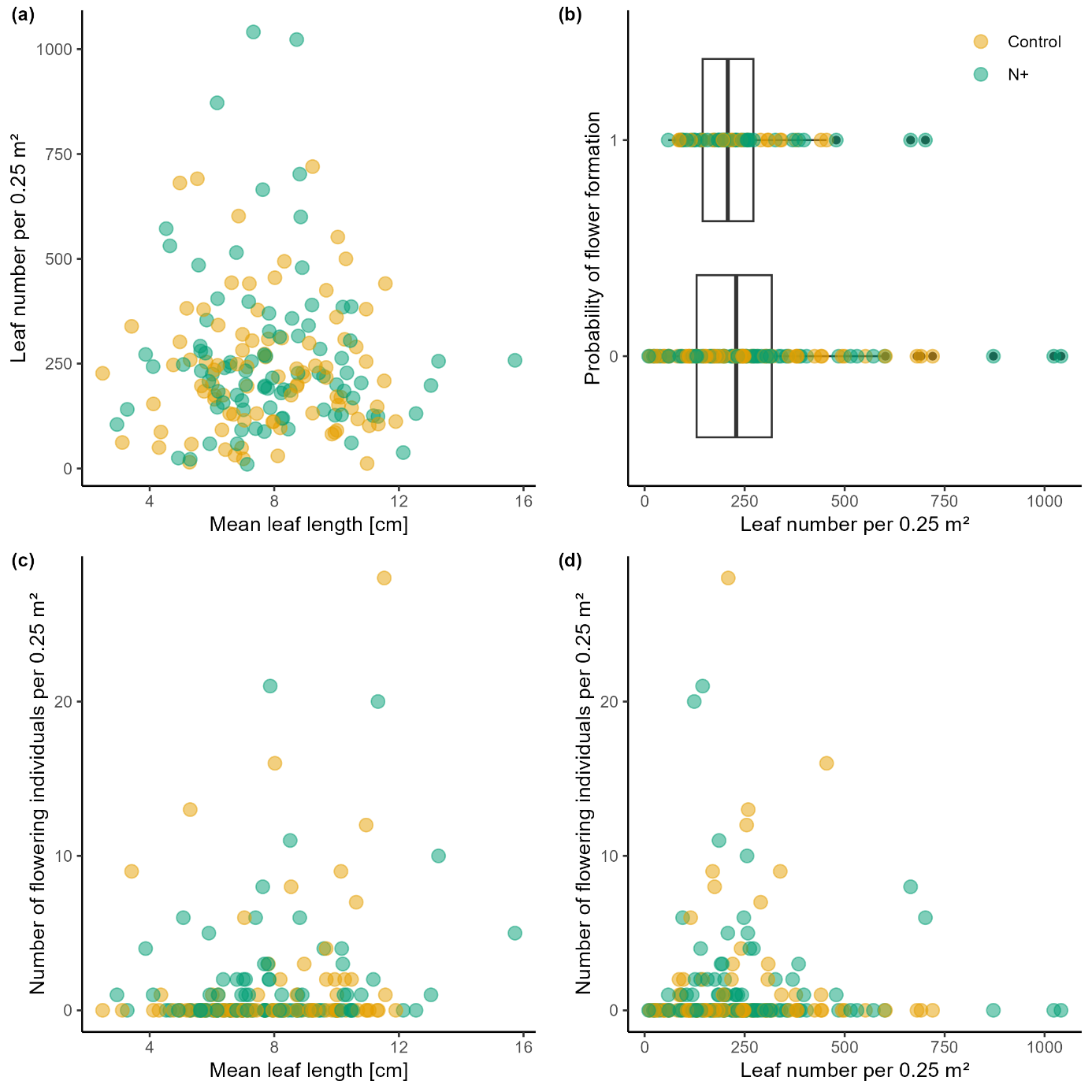


Figure S4 Relationships between population characteristics. **a** leaf density and mean leaf length (Spearman’s r = -0.02, p = 0.749), **b** probability of flower formation and leaf density (Wilcoxon rank sum test: effect size r = 0.02, p = 0.776, **c** number of flowering individuals per plot and mean leaf length (Spearman’s r = 0.18, p = 0.017), **d** number of flowering individuals per plot and leaf density (Spearman’s r = -0.004, p = 0.955).

Table S2 Summary statistics (mean, standard deviation, minimum, maximum) of population characteristics and individual plant traits of *Gagea spathacea*. Leaf density is the number of leaves per 0.25 m²; Δ leaf density is the change in leaf density relative to the density of the previous year; flowering probability is given as number of plots in which flowering individuals were absent or present. Population characteristics were measured annually during the 5-year study period, individual plant traits were measured once at the end of the 5-year study period.

| **Population characteristics** | **Mean** | **SD** | **Min** | **Max** |
| --- | --- | --- | --- | --- |
| Mean leaf length [cm] | 7.87 | 2.23 | 2.49 | 15.73 |
| Leaf density | 251.71 | 174.86 | 10 | 1041 |
| Δ Leaf density | 0.25 | 140.99 | -423 | 629 |
| Number of flowering individuals | 1.57 | 3.87 | 0 | 28 |
|  | **0** | **1** |  |  |
| Flowering probability | 118 | 61 |  |  |
|  |  |  |  |  |
| **Individual traits** | **Mean** | **SD** | **Min** | **Max** |
| Leaf length [cm] | 13.27 | 4.82 | 1.3 | 29.5 |
| Bulb diameter [mm] | 2.60 | 1.06 | 1.13 | 6.11 |
| Leaf dry mass [mg] | 7.28 | 8.93 | 0.51 | 53.40 |
| Bulb dry mass [mg] | 7.28 | 9.99 | 0.35 | 71.30 |
| Leaf N concentration [%] | 3.13 | 0.59 | 1.65 | 5.08 |
| Bulb N concentration [%] | 1.27 | 0.59 | 0.46 | 5.04 |
| Specific leaf N | 0.98 | 0.88 | 0.06 | 7.30 |
| Specific bulb N | 0.69 | 1.17 | 0.02 | 10.15 |
| Leaf δ^13^C | -31.47 | 0.84 | -33.51 | -29.07 |
| Bulb δ^13^C | -31.76 | 0.96 | -33.71 | -28.79 |
| Bulb/leaf size ratio | 0.02 | 0.01 | 0.01 | 0.12 |
| Bulb/leaf N ratio | 0.40 | 0.14 | 0.19 | 1.02 |


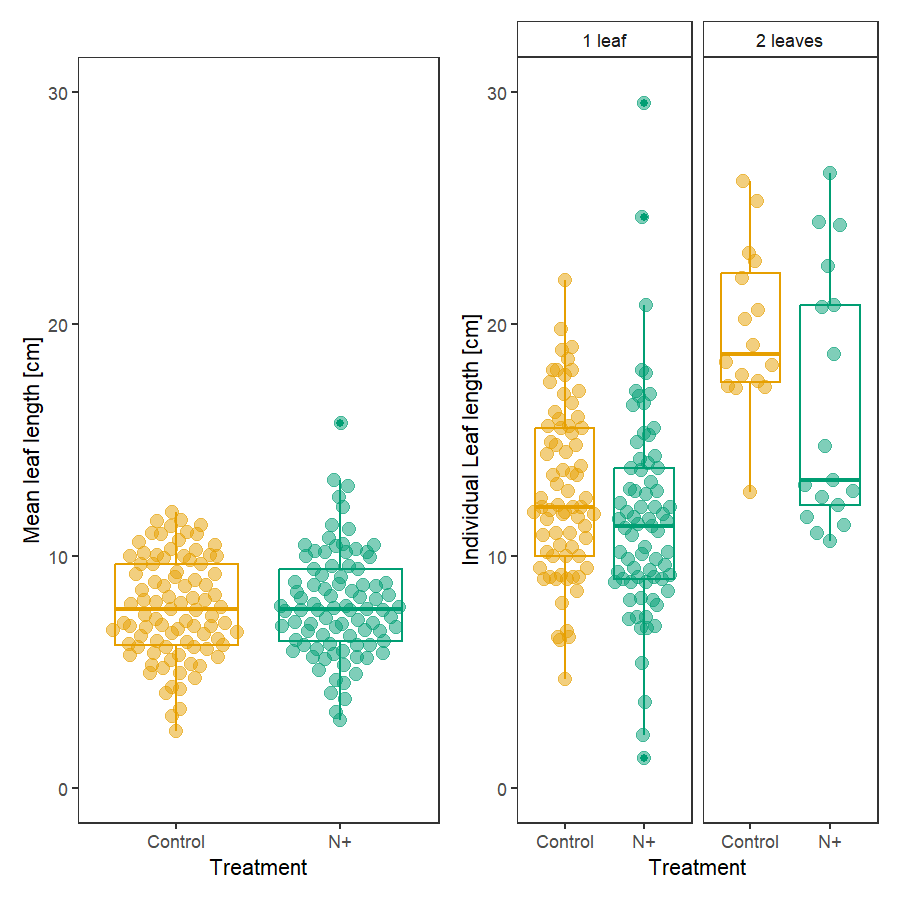


Figure S5 Variation in leaf length of *Gagea spathacea* with nitrogen (N) addition. Left: population level mean leaf length (above-ground leaf length measured in the field from soil surface to leaf tip); right: individual level leaf length (total individual leaf length including the above- and below-ground parts of the leaf measured in the lab from bulb to leaf tip), for one- and for two-leaved plants. Note that it is impossible to distinguish one- and two-leaved individuals in the field in a non-destructive manner. At individual level, the number of leaves per individual determined individual leaf length, but did not show an interaction effect with treatment in the individual level model. Instead, the N treatment only affected individual leaf length in interaction with climate (see Table 2 for model results).
